# Levothyroxine Absorption Test With the Daily Levothyroxine Dose in Patients With “Refractory Hypothyroidism”

**DOI:** 10.1210/jendso/bvaf017

**Published:** 2025-03-04

**Authors:** Philippe Caron, Charlotte Tudor, Solange Grunenwald

**Affiliations:** Department of Endocrinology, Metabolic Diseases and Nutrition, Cardiovascular and Metabolic Unit, CHU Larrey, 31059 Toulouse, France; Department of Endocrinology, Metabolic Diseases and Nutrition, Cardiovascular and Metabolic Unit, CHU Larrey, 31059 Toulouse, France; Department of Endocrinology, Metabolic Diseases and Nutrition, Cardiovascular and Metabolic Unit, CHU Larrey, 31059 Toulouse, France

**Keywords:** hypothyroidism, treatment, levothyroxine, absorption, “Refractory hypothyroidism, ” levothyroxine absorption test

## Abstract

Hypothyroidism is a frequent disease, and oral levothyroxine is the mainstay of its treatment. However, more than 15% of levothyroxine-treated patients fail to achieve the recommended serum TSH level, and “refractory hypothyroidism” is due to either malabsorption, increased metabolism of thyroxine, or nonadherence to treatment. A levothyroxine absorption test must be used to differentiate true malabsorption from nonadherence or pseudo-malabsorption.

We analyzed 166 levothyroxine absorption tests in 143 hypothyroid patients (109 women, mean age 43 ± 1 years) treated with oral levothyroxine. Despite a daily dose of 3.26 ± 0.09 µg/kg/day, mean serum TSH concentration was 25.7 ± 3.7 mU/L. “Refractory hypothyroidism” was in the context of gastritis (24%), *Helicobacter pylori* infection (18%), drug interference with levothyroxine absorption (15.6%), nonadherence to treatment (10%), celiac disease (2.5%), or bariatric surgery (1.2%). After an overnight fast, patients orally took their daily dose of levothyroxine (220 ± 6 µg), and blood samples were drawn before levothyroxine intake and every 2 hours for 24 hours.

After levothyroxine intake, the mean total (basal = 7.64 ± 0.26 µg/dL, peak 9.41 ± 0.28 µg/dL), and free (basal = 12.58 ± 0.42 pg/mL, peak 15.77 ± 0.51 pg/mL) T4 levels increased (*P* < .001), total and free T4 peaks were observed at 4.2 ± 0.23 and 4.30 ± 9.27 hours, respectively. Levothyroxine absorption tests were well tolerated.

In conclusion, in most patients with “refractory hypothyroidism,” this clinical study revealed that the levothyroxine absorption test can be achieved via the absorption of the daily dosage of levothyroxine, and the evaluation of total or free T4 concentrations over 4- or 6-hour follow-up. The test is well tolerated without cardiovascular adverse events.

Hypothyroidism is a frequent endocrine disease and is more common in females and with increasing age. Synthetic levothyroxine is the mainstay of substitutive therapy. After oral administration, low pH in the stomach is essential for optimal dissolution and proper ionization of levothyroxine tablets. Then, levothyroxine absorption takes place primarily in the duodenum and jejunum of the small intestine [1, 2]. In a fasted state, 60% to 80% of the orally administered levothyroxine dose is absorbed, and levothyroxine doses between 1.5 to 1.8 µg/kg/day restore clinical euthyroidism and achieve TSH values in the reference range in most patients with overt primary hypothyroidism. Despite a daily levothyroxine dose of more than 1.9 µg/kg/day, between 15% and 20% of levothyroxine-treated patients present hypothyroid symptoms or have TSH levels above the upper limit of the reference range [3, 4]. Such patients present so called “refractory hypothyroidism” generally because of poor therapeutic compliance, levothyroxine malabsorption syndromes, or accelerated pharmacokinetics of thyroxine [5].

In clinical practice, the levothyroxine absorption test is part of the workup of patients with “refractory hypothyroidism” to differentiate true malabsorption from nonadherence or pseudo-malabsorption [3, 6]. The levothyroxine absorption test must be safe, reliable, easy to perform, minimizing time and the number of blood samples required, and results must be replicable. So far, the levothyroxine absorption test is not standardized [7-18], and multiple protocols have been published with differences regarding the test dose, formulations, test durations, frequency of blood collections, analyte (total T4, free T4), metric (absolute or relative peak or increment), and thresholds for normal absorption.

In this article, we describe the clinical and hormonal results of levothyroxine absorption testing after oral administration of the daily dose of levothyroxine performed in a large cohort of patients with “refractory hypothyroidism.”

## Patients

Patients treated with relatively large levothyroxine doses (≥1.8 µg/kg/day in most patients [94.4%]) with TSH levels above the upper limit of the reference range were admitted in the hospitalization sector of the department of Endocrinology and Metabolic diseases and included in this study.

Before starting levothyroxine absorption testing, a clinical examination was done with:

- History of primary (surgical thyroidectomy, Hashimoto thyroiditis) or central (pituitary adenoma, apoplexy) hypothyroidism and of hormonal substitutive therapy.
- Body weight and height measured for calculation of body mass index (kg/m^2^) and of daily levothyroxine dose (µg/kg/day).
- Evaluation of the factors interfering with levothyroxine absorption or thyroxine metabolism:
  ○ causes of decreased gut absorption: celiac disease, bariatric surgery, jejunoileal bypass, gastrectomy, atrophic or autoimmune gastritis, lactose intolerance, *Helicobacter pylori* infection, inflammatory bowel disease, short bowel syndrome.
  ○ dietary factors interfering with levothyroxine absorption (fiber, grapefruit, soy, coffee).
  ○ drugs reducing levothyroxine absorption when coadministered with levothyroxine tablets: proton pump inhibitors, aluminium hydroxide, sucralfate, ferrous sulphate, calcium carbonate, cholestyramine.
  ○ drugs accelerating the pharmacokinetics of thyroxine, leading to higher dose requirements (rifampicin, phenytoin, phenobarbital, carbamazepine).
- Finally, possible poor therapeutic compliance, low-treatment adherence with missing levothyroxine doses or pseudo-malabsorption by direct patient report.

This work conforms to the Declaration of Helsinki, Good Clinical Practice Guidelines. All patients agreed to participate after the purposes of the levothyroxine absorption testing were explained to them. Patients with abnormal intestinal absorption of levothyroxine had additional tests investigating the underlying causes of levothyroxine malabsorption syndromes or were referred to a gastroenterologist. The study was approved by the scientific board of Département Recherche et Innovation, CHU Toulouse (France) (reference number RnIPH 2024-03).

## Methods

The levothyroxine absorption tests were performed in 143 patients between January 2006 and December 2023. After an overnight fast and with an empty stomach, each patient was given their daily levothyroxine dose, supervised for swallowing of levothyroxine tablets to ensure compliance, and over the next 24 hours to evaluate if the patients presented any side effects.

The first blood sample (baseline) was taken before levothyroxine intake and then they were drawn every 2 hours for a period of 24 hours. Serum total (TT4) and free (FT4) thyroxine concentrations were measured in all samples, and serum TSH levels were evaluated at baseline and in the last blood samples.

In serum samples of the patients, total T4, free T4, and TSH concentrations were analyzed using ADVIA Centaur (Siemens; normal range TT4: 4.5-10.9 µg/dL, FT4: 7.5-16 pg/mL) between 2006 and 2014, and then Cobas 8000 (Roche Diagnostics; normal range TT4: 4.6-11 µg/dL, FT4: 9.3-17 pg/mL) kits between 2015 and 2023, respectively.

After oral administration of the daily dose of levothyroxine, the following data were analyzed:

- Maximum TT4 (µg/dL) and FT4 (pg/mL) concentrations with amplitude of the peak and the timing after levothyroxine intake.
- Increase in TT4 and FT4 concentrations with peak minus baseline concentrations of TT4 and FT4.
- Percentage of levothyroxine absorption [11, 19, 20] calculated with the following equation:
  $$\mathrm{percent}\;\mathrm{absorbed}=\frac{(\mathrm{peak}\;\mathrm{TT}4\text{–}\mathrm{baseline}\;\mathrm{TT}4)(\mu g/\mathrm{dl})\times \mathrm{VD}\times 10}{\mathrm{Total}\;\mathrm{administered}\;\mathrm{levothyroxine}\;\mathrm{dose}\;(g)}$$
  $$\mathrm{Volume}\;\mathrm{of}\;\mathrm{distribution}=0.442\times \mathrm{BMI}\;(\mathrm{kg}/m^{2}).$$

In which BMI indicates body mass index. A percentage of levothyroxine absorption <60% indicated levothyroxine malabsorption.

## Results

### Patients

A total of 143 patients underwent levothyroxine absorption testing: they had 1 (n = 125), 2 (n = 11), or more than 3 (n = 4) levothyroxine absorption tests, respectively, and were included in the study. One hundred and nine patients were female. The mean age of the patients was 43 ± 1 years. Weight and BMI were 69 ± 1 kg and 25.5 ± 0.4 kg/m^2^, respectively.

The causes of hypothyroidism were surgical thyroidectomy for benign (n = 61) or malignant (n = 41) diseases, followed by autoimmune thyroiditis (n = 29), congenital hypothyroidism (n = 5), central hypothyroidism (n = 4), and postradioiodine therapy (n = 3).

At the time of levothyroxine absorption tests, patients were treated with levothyroxine alone (n = 157) or in association with liothyronine (n = 6). The mean dose of levothyroxine was 220 ± 6 µg/day or 3.26 ± 0.09 µg/kg/day and the mean dose of liothyronine was 44 ± 8.2 µg/day. The mean dose of levothyroxine was 207 ± 7 µg/day and 242 ± 13 µg/day after surgery for benign (n = 104) and malignant (n = 39) diseases, respectively, and 205 ± 11 µg/day for autoimmune thyroiditis (n = 29). The mean levothyroxine dose was significantly higher in patients with differentiated thyroid carcinomas (*P* < .02).

At the time of levothyroxine absorption testing, “refractory hypothyroidism” was in the context of autoimmune or atrophic gastritis (n = 40) and *Helicobacter pylori* infection (n = 13) or proton pump inhibitor treatment (n = 5), drug interferences with levothyroxine absorption (proton pump inhibitor n = 11, calcium carbonate n = 5, ferrous sulphate n = 5), *H. pylori* infection (n = 17), gut malabsorption resulting from celiac disease (n = 4) or bariatric surgery (n = 2), and nonadherence or pseudo-malabsorption (n = 17) reported by patients.

### Hormonal data

Before administration of the daily levothyroxine dose, baseline total and free T4 concentrations were 7.64 ± 0.26 µg/dL and 12.58 ± 0.42 pg/mL, respectively. After levothyroxine intake, the maximum total (TT4 = 9.41 ± 0.28 µg/dL, *P* < .001) and free (FT4 = 15.77 ± 0.51 pg/mL, *P* < .001) T4 concentrations increased, and were below the upper limit of the reference range. Before intake of the daily levothyroxine dose, mean baseline TSH level was 25.1 ± 3.3 mIU/L, and 24 hours after levothyroxine intake mean TSH level was not changed (25.5 ± 3.4 mIU/L) (Table 1).

**Table 1.** Total (TT4), free (FT4) thyroxine, and TSH concentrations during levothyroxine absorption test with daily levothyroxine dose in 143 patients with “refractory hypothyroidism”

	Before	Maximum peak	*P* <
TT4 (µg/dL)	7.64 ± 0.26	9.41 ± 0.28	.001
FT4 (pg/mL)	12.58 ± 0.42	15.77 ± 0.51	.001
TSH (mU/L)	25.1 ± 3.3	25.5 ± 3.4	ns
TT4 increase (µg/dL)		1.79 ± 0.08	
FT4 increase (pg/mL)		3.28 ± 0.16	

**Figure 1.**
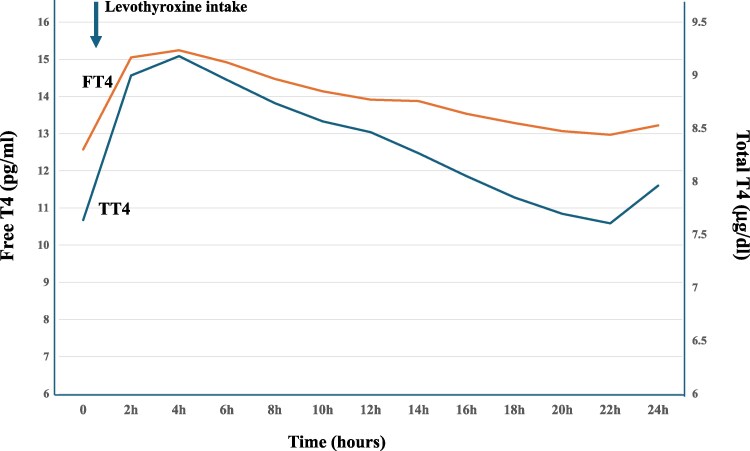
Total (TT4) and free (FT4) thyroxine concentrations after absorption of daily levothyroxine dose in 143 patients with “refractory hypothyroidism.” Normal range TT4: 4.6-11 µg/dL, FT4: 9.3-17 pg/mL.

Total and free T4 levels peaked after 4.2 ± 0.2 hours and 4.3 ± 0.2 hours, respectively. Then, TT4 and FT4 concentrations decreased gradually and returned to baseline values at 22 hours (Fig. 1).

After oral levothyroxine intake, mean increases (maximum minus baseline) of total and free T4 concentrations were 1.79 ± 0.08 µg/dL and 3.28 ± 0.16 pg/mL, respectively (Table 1), and they were correlated with the daily levothyroxine doses (TT4: *P* = .02; FT4: *P* = .01).

The percentage of levothyroxine absorption was less than 100% in 89 patients, and more than 100% in 76 patients (between 100% and 150%: n = 50; >150%: n = 26). Weight and BMI were not higher in patients with a levothyroxine absorption test >100% (weight, 70 ± 19 kg; BMI, 25.8 ± 5.8 kg/m^2^) than in patients with a levothyroxine absorption test of less than 100% (weight, 67 ± 17 kg; BMI, 24.5 ± 5.3 kg/m^2^), and the percentage of levothyroxine absorption was not correlated with the weight and BMI of the patients.

The percentage of levothyroxine absorption decreased from 104 ± 52% in patients with a daily dose of levothyroxine between 2 and 3 µg/kg/day to 81 ± 43% in patients with a daily dose higher than 4 µg/kg/day. The percentage of levothyroxine absorption was negatively correlated with the daily dose of levothyroxine (*P* < .001).

In patients with autoimmune or chronic gastritis, the percentage of levothyroxine absorption was significantly lower in comparison with pseudo-malabsorption patients (60 ± 9% vs 116 ± 19%, *P* < .01) with a significant decrease of total and free thyroxine increases (TT4 *P* < .01; FT4 *P* = .02) (Table 2). Moreover, a decrease of the percentage of levothyroxine absorption as well as of TT4 and FT4 increases was also observed in patients with *H. pylori* infection or proton pump inhibitor treatment (Table 2).

**Table 2.** Percentage of levothyroxine absorption, TT4, and FT4 increases in patients with autoimmune or chronic gastritis, *Helicobacter pylori* infection or proton pump inhibitor therapy during levothyroxine absorption test with daily levothyroxine dose

	n	% absorption	FT4 increase	TT4 increase
Pseudo-malabsorption	12	116 ± 19	3.66 ± 0.40	2.32 ± 0.22
Gastritis	19	60 ± 9*	2.47 ± 0.42**	1.20 ± 0.23*
*Helicobacter pylori*	15	82 ± 9	3.02 ± 0.30	1.58 ± 0.2**
Proton pump inhibitors	9	78 ± 13	2.7 ± 0.34	1.74 ± 0.2

Abbreviations: FT4, free thyroxine; TT4, total thyroxine.

^*^ *P* < .01, ** < .02 vs pseudo-malabsorption group.

### Safety

After oral intake of the daily levothyroxine dose, there were no adverse events reported by the patients during the 24-hour follow-up.

## Discussion

Hypothyroidism is a frequent disease and oral levothyroxine is the mainstay of its life-long replacement treatment. In most patients, synthetic levothyroxine sodium is orally administered: after dissolution and ionization of levothyroxine tablets under acid pH in the stomach, levothyroxine is absorbed in the small intestine (duodenum, jejunum). Plasma thyroxine concentration rises in first 60 to 90 minutes and peaks at 2 hours after levothyroxine intake in euthyroid subjects. In severe hypothyroidism, levothyroxine absorption may be impaired because of edema of the small bowel mucosa; thyroxine concentration peaks at 2 to 3 hours after levothyroxine intake on average.

Most patients with minimal endogenous thyroid function require 1.6 to 1.8 µg/kg/day of levothyroxine sodium to restore clinical euthyroid state and normal TSH concentration in primary hypothyroidism or free T4 concentration in the reference range in central hypothyroidism. In some patients, despite increasing levothyroxine dosage beyond 1.9 µg/kg/day, euthyroid state is not achieved [3, 4]. “Treatment-refractory hypothyroidism” may be due to either poor adherence to levothyroxine treatment or pseudo-malabsorption, the most common cause of treatment-refractory hypothyroidism, increased levothyroxine metabolism by drugs modifying levothyroxine breakdown and gut microbiota affecting the pharmacological homeostasis of administered levothyroxine [1], or true gastrointestinal malabsorption (gastrectomy, bariatric surgery, gastritis) [5]. “Refractory hypothyroidism” is uncommon (9 patients/year have been hospitalized in our department), but data on “treatment-refractory hypothyroidism” are increasingly published. Investigations are warranted to optimize the oral levothyroxine treatment of hypothyroid patients and to correct identifiable underlying causes of “refractory hypothyroidism.”

Classically, to differentiate between noncompliance or pseudo-malabsorption and true malabsorption, levothyroxine absorption testing may be indicated in patients with “refractory hypothyroidism.” Levothyroxine absorption test should be simple, safe, and in an outpatient setting, but the levothyroxine absorption testing is not standardized, and variable doses of levothyroxine have been reported in the literature [7-18, 20]. After high-dose levothyroxine intake, increases of total or free thyroxine concentrations support the idea that a high levothyroxine dose (> 800 µg) may be excessive in some patients [21] and may not be necessary to demonstrate adequate levothyroxine absorption in the majority of patients [11]. On the other hand, a significant increase in plasma thyroxine levels has been observed with lower doses (300-450 µg) of levothyroxine [14]. In our patients, significant increases of total and free thyroxine concentrations were observed (*P* < .001) after intake of the daily dose of levothyroxine, attesting that the daily dose may be sufficient and can be used to evaluate levothyroxine absorption.

After oral levothyroxine intake, thyroxine levels typically rise in 1 hour and the peak is within the first 3 hours [17]. Measurements of serum thyroxine concentrations 3 to 4 hours after levothyroxine intake can be used to show normal levothyroxine absorption. In our clinical study with 2-hour blood sampling, total and free thyroxine peaks were observed at 4.2 ± 0.2 hours and 4.3 ± 0.2 hours, respectively. Interestingly, only 4 results after the 6-hour levothyroxine absorption test were discordant (2.4%) when hormonal follow-up was prolonged for 24 hours. Therefore, to minimize time, number of blood samples and reduce the cost, the levothyroxine absorption test could be reduced to 4 to 6 hours with hourly or 2-hour blood sampling for hormonal evaluation.

Theoretically, the ingestion of a high dose (800-2000 µg) of levothyroxine may be associated with side effects such as palpitations, angina, or cardiac arrhythmia in a small proportion of patients with lower weight, elderly patients, or those with heart diseases (cardiac arrythmia and coronary artery disease) [20, 22]. However, these adverse effects are rarely observed [23] because changes in thyroxine levels are minimal and they remain within the reference range in most patients [24], and T4 is bound by thyroxine-binding globulin and slowly enters the tissues, and also free T4 needs to be converted to T3 first to be biologically active. In our hypothyroid patients with mean TSH concentrations >25 mU/L, no clinical side effects were reported during and the 24-hour follow-up after levothyroxine absorption testing, and mean total or free thyroxine concentration peaks were below the upper limit of the reference range. So, our clinical study shows that the levothyroxine absorption testing with a daily dose of levothyroxine is safe and can be used to prevent the rare cardiovascular side effects observed with high doses of levothyroxine.

In most studies, levothyroxine absorption testing is considered normal after absorption of a high dose of levothyroxine [5, 20] if the 4-hour peak concentration of total thyroxine exceeds 6 µg/dL, the 4-hour peak concentration of free thyroxine is greater than 0.40 ng/dL, or if the percentage absorption of levothyroxine is greater than 60%. However, using total thyroxine concentration in the hormonal results or the calculation of percentage absorption of levothyroxine appears to be a limitation, as total thyroxine levels are more greatly influenced by intrinsic and extrinsic factors when compared to free thyroxine levels, and free thyroxine concentrations are now routinely measured as a first-line test instead of total thyroxine in clinical practice. Total and free thyroxine concentrations are correlated highly in patients with severe hypothyroidism [16], as well as in our patients (*P* < .001), so free thyroxine levels can be used interchangeably with total thyroxine levels in quantitative and qualitative levothyroxine absorption testing [16]. More studies are needed to confirm that FT4 concentrations can replace TT4 levels in the hormonal evaluation of levothyroxine absorption testing with a daily dose of levothyroxine. Moreover, a significant reduction in TSH concentrations within 2 hours of oral levothyroxine intake has been reported in a previous study [24], but in our patients, mean TSH levels did not decline 24 hours after oral levothyroxine administration. So, TSH measurements did not provide additional information in this levothyroxine absorption test [21].

It is well known that gastric acid pH plays an important role in levothyroxine absorption, and an increased gastric pH decreases the solubility of levothyroxine tablets and can potentially impair absorption of orally administered levothyroxine sodium [2, 5, 25, 26]. In our patients with autoimmune or atrophic gastritis, gastrointestinal diseases (*H. pylori* infection) or proton pump inhibitor treatment, levothyroxine absorption was decreased (Table 2). Nevertheless, more studies are necessary to define absolute and/or relative normal increases of total T4 and free T4 parameters in order to differentiate true malabsorption from nonadherence or pseudo-malabsorption, and before this levothyroxine absorption test could be implemented broadly in clinical practice.

The study has limitations: first, the retrospective design resulted in inherent bias with the method. Second, the authors do not determine sensitivity and specificity of the cutoff total and FT4 values to characterize the well-established gold standard and differentiate between malabsorption syndrome and noncompliance (manuscript in preparation).

The strengths of this clinical study are that:

- the large number of patients included and explored with this levothyroxine absorption testing constitutes the largest cohort of patients with refractory hypothyroidism evaluated so far.
- the daily dose used during the levothyroxine absorption testing. In the literature, most studies used a high dose (1000 µg) of levothyroxine with potential and rare side effects. In our study, there were no adverse events reported by the patients during the 24-hour follow-up. Therefore, levothyroxine absorption testing with daily dosing of levothyroxine can be used especially in patients with lower weight, elderly patients, and patients with heart disease. As previously considered [11], the doses of levothyroxine administered in most studies are higher than what would be needed to demonstrate adequate absorption on the basis of total or free T4 changes.

## Data Availability

All datasets generated during and/or analyzed during the current study are not publicly available but are available from the corresponding author on reasonable request.

## Abbreviations

BMI: body mass index
FT4: free thyroxine
TT4: free thyroxine
