# Correction to: “Levothyroxine Absorption Test With the Daily Levothyroxine Dose in Patients With ‘Refractory Hypothyroidism’”

**DOI:** 10.1210/jendso/bvaf076

**Published:** 2025-05-16

In the above-named article by Caron P, Tudor C, and Grunenwald S (*J Endo Society*. 2025; 9(4); doi: 10.1210/jendso/bvaf017), there were errors in units of measurement for levothyroxine doses and measurements in the abstract, in the text, in Table 1, and in the caption to Figure 1.

In the originally published article, in second paragraph of the abstract, the second sentence read “Despite a daily dose of 3.26 ± 0.09 g/kg/day, mean serum TSH concentration was 25.7 ± 3.7 mU/L.” The letter “µ” was accidentally excluded, incorrectly making the unit grams instead of micrograms as intended. Therefore, “g/kg/day” has been replaced with “µg/kg/day.” In the corrected article, the sentence now reads “Despite a daily dose of 3.26 ± 0.09 µg/kg/day, mean serum TSH concentration was 25.7 ± 3.7 mU/L.”

In the originally published article, in second paragraph of the abstract, the fourth sentence read “After an overnight fast, patients orally took their daily dose of levothyroxine (220 ± 6 g), and blood samples were drawn before levothyroxine intake and every 2 hours for 24 hours.” To change grams to micrograms, “g” has been replaced with “µg.” In the corrected article, the sentence now reads “After an overnight fast, patients orally took their daily dose of levothyroxine (220 ± 6 µg), and blood samples were drawn before levothyroxine intake and every 2 hours for 24 hours.”

In the originally published article, in third paragraph of the abstract, the first sentence read “After levothyroxine intake, the mean total (basal = 7.64 ± 0.26 g/dL, peak 9.41 ± 0.28 g/dL), and free (basal = 12.58 ± 0.42 pg/mL, peak 15.77 ± 0.51 pg/mL) T4 levels increased (*P* < .001), total and free T4 peaks were observed at 4.2 ± 0.23 and 4.30 ± 9.27 hours, respectively.” To change grams to micrograms, “g/dL” has been replaced with “µg/dL.” In the corrected article, the sentence now reads “After levothyroxine intake, the mean total (basal = 7.64 ± 0.26 µg/dL, peak 9.41 ± 0.28 µg/dL), and free (basal = 12.58 ± 0.42 pg/mL, peak 15.77 ± 0.51 pg/mL) T4 levels increased (*P* < .001), total and free T4 peaks were observed at 4.2 ± 0.23 and 4.30 ± 9.27 hours, respectively.”

In the originally published article, in the first paragraph, the fifth and sixth sentences read “In a fasted state, 60% to 80% of the orally administered levothyroxine dose is absorbed, and levothyroxine doses between 1.5 to 1.8 g/kg/day restore clinical euthyroidism and achieve TSH values in the reference range in most patients with overt primary hypothyroidism. Despite a daily levothyroxine dose of more than 1.9 g/kg/day, between 15% and 20% of levothyroxine-treated patients present hypothyroid symptoms or have TSH levels above the upper limit of the reference range [3, 4].” To change grams to micrograms, “g/kg/day” has been replaced with “µg/kg/day.” In the corrected article, the sentences now read “In a fasted state, 60% to 80% of the orally administered levothyroxine dose is absorbed, and levothyroxine doses between 1.5 to 1.8 µg/kg/day restore clinical euthyroidism and achieve TSH values in the reference range in most patients with overt primary hypothyroidism. Despite a daily levothyroxine dose of more than 1.9 µg/kg/day, between 15% and 20% of levothyroxine-treated patients present hypothyroid symptoms or have TSH levels above the upper limit of the reference range [3, 4].”

In the originally published article, in the Patients section, the first paragraph read “Patients treated with relatively large levothyroxine doses (≥1.8 g/kg/day in most patients [94.4%]) with TSH levels above the upper limit of the reference range were admitted in the hospitalization sector of the department of Endocrinology and Metabolic diseases and included in this study.” To change grams to micrograms, “g/kg/day” has been replaced with “µg/kg/day.” In the corrected article, the paragraph now reads “Patients treated with relatively large levothyroxine doses (≥1.8 µg/kg/day in most patients [94.4%]) with TSH levels above the upper limit of the reference range were admitted in the hospitalization sector of the department of Endocrinology and Metabolic diseases and included in this study.”

In the originally published article, in the second paragraph of the Patients section, the second bullet point read “Body weight and height measured for calculation of body mass index (kg/m^2^) and of daily levothyroxine dose (g/kg/day).” To change grams to micrograms, “g/kg/day” has been replaced with “µg/kg/day.” In the corrected article, the sentence now reads “Body weight and height measured for calculation of body mass index (kg/m^2^) and of daily levothyroxine dose (µg/kg/day).”

In the originally published article, in the Methods section, the third paragraph read “In serum samples of the patients, total T4, free T4, and TSH concentrations were analyzed using ADVIA Centaur (Siemens; normal range TT4: 4.5-10.9 mg/dL, FT4: 7.5-16 pg/mL) between 2006 and 2014, and then Cobas 8000 (Roche Diagnostics; normal range TT4: 4.6-11 mg/dL, FT4: 9.3-17 pg/mL) kits between 2015 and 2023, respectively.” The letter “µ” was accidentally replaced with “m,” incorrectly making the unit milligrams instead of micrograms as intended, so “mg/dL” has been replaced with “µg/dL.” In the corrected article, the paragraph now reads “In serum samples of the patients, total T4, free T4, and TSH concentrations were analyzed using ADVIA Centaur (Siemens; normal range TT4: 4.5-10.9 µg/dL, FT4: 7.5-16 pg/mL) between 2006 and 2014, and then Cobas 8000 (Roche Diagnostics; normal range TT4: 4.6-11 µg/dL, FT4: 9.3-17 pg/mL) kits between 2015 and 2023, respectively.”

In the originally published article, in the fourth paragraph of the Methods section, the first bullet point read “Maximum TT4 (g/dL) and FT4 (pg/mL) concentrations with amplitude of the peak and the timing after levothyroxine intake.” To change grams to micrograms, “g/dL” has been replaced with “µg/dL.” In the corrected article, the sentence now reads “Maximum TT4 (µg/dL) and FT4 (pg/mL) concentrations with amplitude of the peak and the timing after levothyroxine intake.”

In the originally published article, in the fourth paragraph of the Methods section, in the third bullet point, the top line of the right-hand term of the equation contained “g/dl.” To change grams to micrograms, “g/dl” has been replaced with “µg/dl” in the corrected article.

In the originally published article, in the Patients subsection of the Results section, in the third paragraph, the second and third sentences read “The mean dose of levothyroxine was 220 ± 6 g/day or 3.26 ± 0.09 g/kg/day and the mean dose of liothyronine was 44 ± 8.2 g/day. The mean dose of levothyroxine was 207 ± 7 g/day and 242 ± 13 g/day after surgery for benign (n = 104) and malignant (n = 39) diseases, respectively, and 205 ± 11 g/day for autoimmune thyroiditis (n = 29).” To change grams to micrograms, “g/day” has been replaced with “µg/day,” and “g/kg/day” has been replaced with “µg/kg/day.” In the corrected article, the sentences now read “The mean dose of levothyroxine was 220 ± 6 µg/day or 3.26 ± 0.09 µg/kg/day and the mean dose of liothyronine was 44 ± 8.2 µg/day. The mean dose of levothyroxine was 207 ± 7 µg/day and 242 ± 13 µg/day after surgery for benign (n = 104) and malignant (n = 39) diseases, respectively, and 205 ± 11 µg/day for autoimmune thyroiditis (n = 29).”

In the originally published article, in the Hormonal Data subsection of the Results section, in the first paragraph, the first and second sentences read “Before administration of the daily levothyroxine dose, baseline total and free T4 concentrations were 7.64 ± 0.26 g/dL and 12.58 ± 0.42 pg/mL, respectively. After levothyroxine intake, the maximum total (TT4 = 9.41 ± 0.28 g/dL, *P* < .001) and free (FT4 = 15.77 ± 0.51 pg/mL, *P* < .001) T4 concentrations increased, and were below the upper limit of the reference range.” To change grams to micrograms, “g/dL” has been replaced with “µg/dL.” In the corrected article, the sentences now read “Before administration of the daily levothyroxine dose, baseline total and free T4 concentrations were 7.64 ± 0.26 µg/dL and 12.58 ± 0.42 pg/mL, respectively. After levothyroxine intake, the maximum total (TT4 = 9.41 ± 0.28 µg/dL, *P* < .001) and free (FT4 = 15.77 ± 0.51 pg/mL, *P* < .001) T4 concentrations increased, and were below the upper limit of the reference range.”

In the originally published article, in the Hormonal Data subsection of the Results section, the third paragraph read “After oral levothyroxine intake, mean increases (maximum minus baseline) of total and free T4 concentrations were 1.79 ± 0.08 g/dL and 3.28 ± 0.16 pg/mL, respectively (Table 1), and they were correlated with the daily levothyroxine doses (TT4: P = .02; FT4: *P* = .01).” To change grams to micrograms, “g/dL” has been replaced with “µg/dL.” In the corrected article, the paragraph now reads “After oral levothyroxine intake, mean increases (maximum minus baseline) of total and free T4 concentrations were 1.79 ± 0.08 µg/dL and 3.28 ± 0.16 pg/mL, respectively (Table 1), and they were correlated with the daily levothyroxine doses (TT4: P = .02; FT4: *P* = .01).”

In the originally published article, in the Hormonal Data subsection of the Results section, in the fifth paragraph, the first sentence read “The percentage of levothyroxine absorption decreased from 104 ± 52% in patients with a daily dose of levothyroxine between 2 and 3 g/kg/day to 81 ± 43% in patients with a daily dose higher than 4 g/kg/day.” To change grams to micrograms, “g/kg/day” has been replaced with “µg/kg/day.” In the corrected article, the sentence now reads “The percentage of levothyroxine absorption decreased from 104 ± 52% in patients with a daily dose of levothyroxine between 2 and 3 µg/kg/day to 81 ± 43% in patients with a daily dose higher than 4 µg/kg/day.”

In the originally published article, in the second paragraph of the Discussion section, the first and second sentences read “Most patients with minimal endogenous thyroid function require 1.6 to 1.8 g/kg/day of levothyroxine sodium to restore clinical euthyroid state and normal TSH concentration in primary hypothyroidism or free T4 concentration in the reference range in central hypothyroidism. In some patients, despite increasing levothyroxine dosage beyond 1.9 g/kg/day, euthyroid state is not achieved [3, 4].” To change grams to micrograms, “g/kg/day” has been replaced with “µg/kg/day.” In the corrected article, the sentences now read “Most patients with minimal endogenous thyroid function require 1.6 to 1.8 µg/kg/day of levothyroxine sodium to restore clinical euthyroid state and normal TSH concentration in primary hypothyroidism or free T4 concentration in the reference range in central hypothyroidism. In some patients, despite increasing levothyroxine dosage beyond 1.9 µg/kg/day, euthyroid state is not achieved [3, 4].”

In the originally published article, in the third paragraph of the Discussion section, the third and fourth sentences read “After high-dose levothyroxine intake, increases of total or free thyroxine concentrations support the idea that a high levothyroxine dose (> 800 g) may be excessive in some patients [21] and may not be necessary to demonstrate adequate levothyroxine absorption in the majority of patients [11]. On the other hand, a significant increase in plasma thyroxine levels has been observed with lower doses (300-450 g) of levothyroxine [14].” To change grams to micrograms, “g” has been replaced with “µg.” In the corrected article, the sentences now read “After high-dose levothyroxine intake, increases of total or free thyroxine concentrations support the idea that a high levothyroxine dose (> 800 µg) may be excessive in some patients [21] and may not be necessary to demonstrate adequate levothyroxine absorption in the majority of patients [11]. On the other hand, a significant increase in plasma thyroxine levels has been observed with lower doses (300-450 µg) of levothyroxine [14].”

In the originally published article, in the fifth paragraph of the Discussion section, owing to a drafting error, the first sentence read “Theoretically, the ingestion of a high dose (800-2000 mg) of levothyroxine may be associated with side effects such as palpitations, angina, or cardiac arrhythmia in a small proportion of patients with lower weight, elderly patients, or those with heart diseases (cardiac arrythmia and coronary artery disease) [20, 22].” To change milligrams to micrograms, “mg” has been replaced with “µg.” In the corrected article, the sentence now reads “Theoretically, the ingestion of a high dose (800-2000 µg) of levothyroxine may be associated with side effects such as palpitations, angina, or cardiac arrhythmia in a small proportion of patients with lower weight, elderly patients, or those with heart diseases (cardiac arrythmia and coronary artery disease) [20, 22].”

In the originally published article, in the sixth paragraph of the Discussion section, the first sentence read “In most studies, levothyroxine absorption testing is considered normal after absorption of a high dose of levothyroxine [5, 20] if the 4-hour peak concentration of total thyroxine exceeds 6 g/dL, the 4-hour peak concentration of free thyroxine is greater than 0.40 ng/dL, or if the percentage absorption of levothyroxine is greater than 60%.” To change grams to micrograms, “g/dL” has been replaced with “µg/dL.” In the corrected article, the sentence now reads “In most studies, levothyroxine absorption testing is considered normal after absorption of a high dose of levothyroxine [5, 20] if the 4-hour peak concentration of total thyroxine exceeds 6 µg/dL, the 4-hour peak concentration of free thyroxine is greater than 0.40 ng/dL, or if the percentage absorption of levothyroxine is greater than 60%.”

In the originally published article, in the ninth paragraph of the Discussion section, in the second bullet point, owing to a drafting error, the second sentence read “In the literature, most studies used a high dose (1000 mg) of levothyroxine with potential and rare side effects.” To change milligrams to micrograms, “mg” has been replaced with “µg.” In the corrected article, the sentence now reads “In the literature, most studies used a high dose (1000 µg) of levothyroxine with potential and rare side effects.”

In the originally published article, in leftmost column of Table 1, the first row cell read “TT4 (g/dL),” and the fourth row cell read “TT4 increase (g/dL).” To change grams to micrograms, “g/dL” has been replaced with “µg/dL.” In the corrected article, the first row cell now reads “TT4 (µg/dL),” and the fourth row cell now reads “TT4 increase (µg/dL).”

In the originally published article, the second sentence of the caption to Figure 1 read “Normal range TT4: 4.6-11 g/dL, FT4 9.3-17 pg/ml.” To change grams to micrograms, “g/dL” has been replaced with “µg/dL.” In the corrected article, the second sentence of the caption to Figure 1 reads “Normal range TT4: 4.6-11 µg/dL, FT4 9.3-17 pg/ml.”

The publisher acknowledges the errors caused by the omission of the symbol µ.

The article has been corrected online.

doi: 10.1210/jendso/bvaf017

